# Composition and temperature dependence of self-diffusion in Si_1−*x*_Ge_*x*_ alloys

**DOI:** 10.1038/s41598-017-01301-6

**Published:** 2017-05-02

**Authors:** Vassilis Saltas, Alexander Chroneos, Filippos Vallianatos

**Affiliations:** 1 School of Applied Sciences, Technological Educational Institute of Crete, Crete, Greece; 2 Department of Materials, Imperial College London, London, SW7 2AZ United Kingdom; 3 Faculty of Engineering, Environment and Computing, Coventry University, Priory Street, Coventry, CV1 5FB United Kingdom

## Abstract

The knowledge of diffusion processes in semiconducting alloys is very important both technologically and from a theoretical point of view. Here we show that, self-diffusion in Si_1−*x*_Ge_*x*_ alloys as a function of temperature and Ge concentration can be described by the *cBΩ* thermodynamic model. This model connects the activation Gibbs free energy of point defects formation and migration with the elastic and expansion properties of the bulk material. The approach allows the systematic investigation of point defect thermodynamic parameters such as activation enthalpy, activation entropy and activation volume, based on the thermo-elastic properties (bulk modulus and its derivatives, mean atomic volume and thermal expansion coefficient) of the two end-members of the Si_1−*x*_Ge_*x*_ alloy. Considerable deviations from Vegard’s law are observed, due to the diversification of the bulk properties of Si and Ge, in complete agreement with the available experimental data.

## Introduction

Over the past years in microelectronics there was the technological drive to replace silicon (Si) with higher mobility substrates such as silicon germanium (Si_1−*x*_Ge_*x*_) alloys or germanium (Ge). These materials have some common features to Si, however, their defect processes differ and were not as well established as in Si^1–9^. For example, although Ge is isostructural to Si its defect processes are very different and this consitutes the formation of *n*-type doped Ge regions problematic (high *n*-type dopant diffusion)^8^. Considering Si_1−*x*_Ge_*x*_ it can be described as a group IV semiconductor random alloy as effectively there is one lattice site but two atomic species that can occupy it. Therefore, in Si_1−*x*_Ge_*x*_ there is a range of local environments including Si-rich and Ge-rich regions that can influence defect processes such as the formation of dopant-defect clusters and self-diffusion^10^. From an experimental viewpoint self-diffusion in Si_1−*x*_Ge_*x*_ has been studied for numerous years^11–13^, whereas the increasing computational resources and the use of density functional theory (DFT) over the past years have facilitated the application of theoretical approaches to study Si_1−*x*_Ge_*x*_^14, 15^. Although DFT can provide insights into the diffusion properties of ordered materials it is more difficult to implement when considering random alloys as this will require numerous large cells. Even the use of methods such as special quasirandom structures, which can constitute most random alloy issues computational tractable will require extensive resources when considering the complete composition and temperature range for random alloys, given that ab initio molecular dynamic calculations will be required to study diffusion.

Thermodynamic approaches may bridge this gap as they can be employed in synergy to experiment or advanced computational modeling. Such a well-established thermodynamic model is the so-called *cBΩ* model, which has been employed during the last four decades (e.g., see ref. 16) to describe the point defect thermodynamic parameters in numerous materials, including metals, oxides, semiconductors alkali and silver halides, diamond and minerals of geophysical interest, as well as in materials that exhibit superionic conductivity at high temperatures^17–27^. The model is based on the theoretical justification that the activation Gibbs free energy of the formation (or migration, or activation) of a point defect is proportional to the bulk modulus of the solid material, *B* and its mean atomic volume, *Ω*, i.e., *g* ^*i*^ = *c* ^*i*^ *BΩ*, where *i* refers to the formation, migration or activation process^28–30^.

The significance of the *cBΩ* model to describe successfully the point defect thermodynamic parameters in different categories of solids has recently emerged with its implementation in semiconductors (Si, Ge, GaAs) and nuclear fuels^31–37^. The potential of the model can be also extended to describe self- or hetero-diffusion in alloys but the examples are rather limited and are restricted only to alkali- or silver-halides mixed crystals and mixed-oxide nuclear fuels^37–40^. In the present study, self-diffusion in Si_1−*x*_Ge_*x*_ is investigated by employing the *cBΩ* thermodynamic model, in conjunction with recent experimental results. Based on the bulk properties of the two end-members, various point defect thermodynamic parameters, such as activation enthalpy, activation entropy and activation volume have been calculated as a function of temperature and Ge concentration.

## Results and Discussion

### Determining the Thermo-elastic properties of the Si_1−*x*_Ge_*x*_ alloys

To describe self-diffusion in a binary alloy A_1−*x*_B_*x*_ (such as Si_1−*x*_Ge_*x*_), the *cBΩ* thermodynamic model can be applied by considering that, each atom of the component B that is added to the homogeneous crystal of the pure component A can be treated as a point defect^30^. The various point defect thermodynamic parameters, such as activation Gibbs free energy *g* ^*act*^, activation enthalpy *h* ^*act*^, activation entropy *s* ^*act*^ and activation volume *υ* ^*act*^, can be expressed through the same relations, as in the case of a unary solid (refer to Eqs 7 and 9–11 of Methods), where the thermo-elastic properties, i.e., the bulk modulus, B, its derivatives (∂*B*/∂*T*|_*P*_ and ∂*B*/∂*P*|_*T*_) and the volume thermal expansion coefficient, *β* refer to the alloy and are functions of temperature and the molar concentration, *x*. To estimate these bulk properties for the Si_1−*x*_Ge_*x*_ alloy, from the corresponding properties of the two constituents (Si and Ge), we proceed as follows.

The composition and temperature dependence of the molar volume of the Si_1−*x*_Ge_*x*_ alloy may be expressed to a first approximation according to the following relation

1 $${V}_{S{i}_{1-x}G{e}_{x}}(x,T)=(1-x){V}_{Si}(T)+x{V}_{Ge}(T)$$

where *x* is the molar concentration of Ge in the alloy and *V*_*Si*_, *V*_*Ge*_ denote the molar volumes of Si and Ge respectively. The derivation of the above equation is based on the assumption that the volume change of the alloy due to the replacement of one atom of the constituent A with an atom of constituent B is independent of the composition, *x* ^30^. In Eq. 1 we may substitute the molar volumes *V*_i_ with the mean atomic volumes *Ω*_i_ of the alloy and its end-members. Considering that the lattice constant of the Si_1−*x*_Ge_*x*_ alloy deviates slightly from Vegard’s law^41, 42^, Eq. 1 may be used as a first approximation to estimate the mean atomic volume of the alloy, as a function of concentration and temperature (for more details refer to the Supplementary Information).

The composition (and temperature) dependence of the bulk modulus, ${B}_{S{i}_{1-x}G{e}_{x}}$ is estimated from the pressure derivative of the molar volume of the alloy, as given by Eq. 1. Recalling that *B* = −*V*(∂*P*/∂*V*|_*T*_):

2 $${B}_{S{i}_{1-x}G{e}_{x}}(x,T)=\frac{1+x({\Omega }_{Ge}/{\Omega }_{Si}-1)}{1+x(\frac{{\Omega }_{Ge}/{\Omega }_{Si}}{{B}_{Ge}/{B}_{Si}}-1)}{B}_{Si}$$

where we have replaced the ratio of the molar volumes of the two end members (*V*_*Ge*_/*V*_*Si*_) with the ratio of their mean atomic volumes (*Ω*_*Ge*_/*Ω*_*Si*_). This non-linear equation in *x* provides a direct estimation of the bulk modulus of the Si_1−*x*_Ge_*x*_ alloy, at any desired concentration and temperature, which is based solely on the bulk properties of the end members^30^. Regarding Ge, the available data of *B*_*Ge*_(*T*) and *Ω*_*Ge*_(*T*) suggest linear relations with respect to temperature, i.e., *B*_*Ge*_ = *B*_*o*_ + (*T* − *T*_*o*_)(∂*B*/∂*T*)_*P*_ and *Ω*_*Ge*_(*T*) = *Ω*_*o*_[1 + *β*_*o*_(*T* − *T*_*o*_)], where the subscript refers to the corresponding properties at room temperature^32^. These values^43–46^ are summarized in Table 1. For Si, the mean atomic volume, *Ω*_*Si*_(*T*) was estimated from the lattice parameter of the Si crystal structure, ${\rm{a}}(T)={{\rm{a}}}_{o}(1+{\int }_{{T}_{o}}^{T}a(T)dT)$, where a_*o*_ denotes the lattice constant at *T*_*o*_. The linear thermal expansion coefficient, *a*(*T*) is simplified to a linear relation in the temperature range (963 K–1543 K) of the present study (refer to Table 1)^33, 47–49^. A 2nd order polynomial fitting has been used to describe the bulk modulus, *B*(*T*) of Si, at this temperature range, as it has been recently reported^33^.

**Table 1:** List of necessary properties of the two end members (Si and Ge) for the proper implementation of the *cBΩ* model in Si_1−*x*_Ge_*x*_ alloys at different Ge concentrations.

Property (units)	Silicon	Ref. or calculating method	Germanium	Ref. or calculating method
Mean atomic volume, *Ω* (m^3^)	20.02 × 10^−30^ (300 K, *P* = 0) 20.16 × 10^−30^ (at 963 K)	Calculated from *β*	22.64 × 10^−30^ (295 K, *P* = 0)	ref. 43
Bulk modulus, *B* (GPa)	91.9 (293 K) 87.0 (963 K)	ref. 44	74.9 (295 K)	ref. 44
(∂*B*/∂*P*)_*T*_	5.08 (300 K, P = 0)	ref. 45	3.0 (295 K, *P* = 0)	ref. 44
(∂*B*/∂*P*)_*P*_ (PaK^−1^)	0.0242 × 10^9^ − 3.46 × 10^4^ *T*	Derived from fitting of *B*(*Τ*) at high *T*	−0.0126 × 10^9^	ref. 45
Coefficient of volume thermal expansion, *β* (K^−1^)	3(3.725 (1 − *e* ^−5.88 × 10−3(*T* − 124)^) + 5.548 × 10^−4^ *T*) × 10^−6^ (120 K–1500 K) (1.13 + 1.89 × 10^−4^ *T*) × 10^−5^ (963 K–1543 K)	ref. 46	1.82 × 10^−5^ + 1.03 × 10^−7^ (*T* − 273) − 1.05 × 10^−12^ (*T* − 273)^2^	ref. 46
${(\partial \beta /\partial {\rm T})}_{P}$ (K^−2^)	1.89 × 10^−9^ (963 K–1543 K)	Derived from the analytical expression of *β*	1.03 × 10^−7^ − 2.10 × 10^−12^ (*T* − 273)	Derived from the analytical expression of *β*

**Figure 1:**
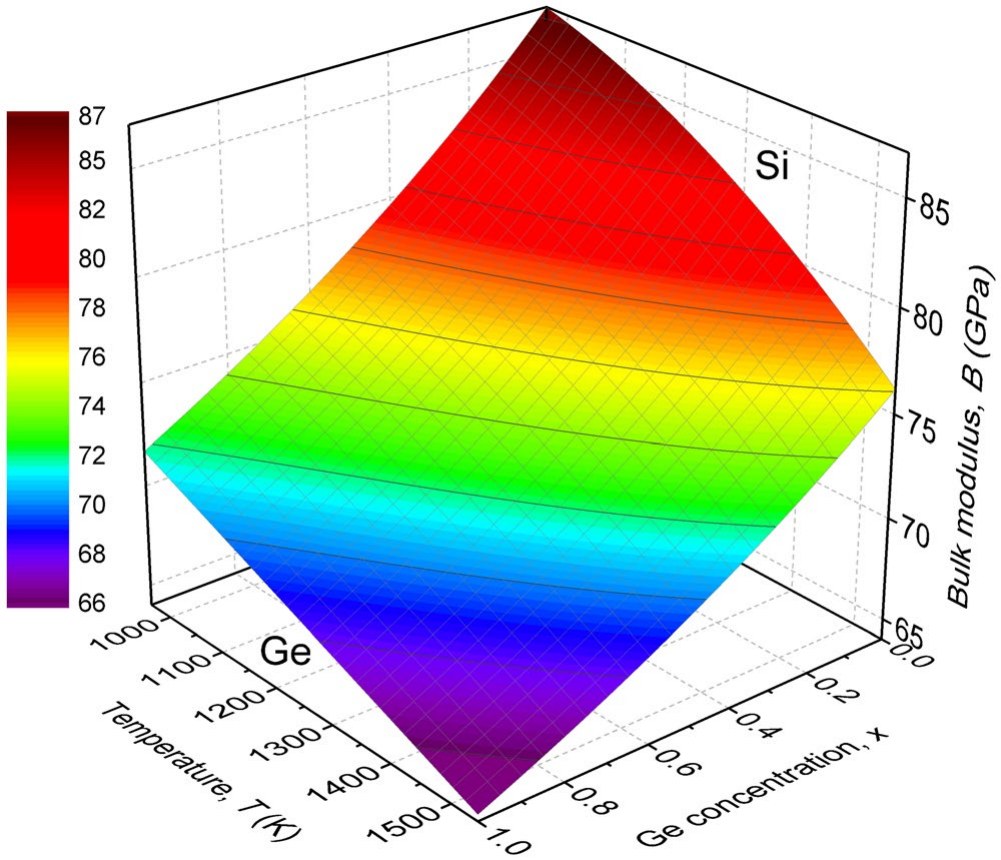
3D surface plot of the bulk modulus of Si_1−*x*_Ge_*x*_ alloys as a function of temperature and Ge concentration, calculated from Eq. 2.

Based on the previous considerations, the 3D surface plot of the bulk modulus, ${B}_{S{i}_{1-x}G{e}_{x}}$ as a function of temperature and Ge concentration is depicted in Fig. 1. The temperature range is restricted to 963 K–1543 K, where diffusion measurements of Si and Ge in Si_1−*x*_Ge_*x*_ alloys have been reported and are used in the present study^2, 9^.

The isobaric temperature derivative of the bulk modulus of the alloy, $\partial {B}_{S{i}_{1-x}G{e}_{x}}/\partial T{|}_{P}$, is obtained by differentiating Eq. 2, which finally gives

3 $$\begin{array}{l}{\frac{\partial {B}_{S{i}_{1-x}G{e}_{x}}(x)}{\partial T}|}_{P}=\frac{1+x({\Omega }_{Ge}/{\Omega }_{Si}-1)}{1+x(\frac{{\Omega }_{Ge}/{\Omega }_{Si}}{{B}_{Ge}/{B}_{Si}}-1)}{\frac{\partial {B}_{Si}}{\partial T}|}_{P}\\ +\,{B}_{Si}\frac{(1-x)\frac{{\Omega }_{Ge}}{{\Omega }_{Si}}({\beta }_{Ge}-{\beta }_{Si})[1-x\frac{{B}_{Si}}{{B}_{Ge}}(1-\frac{{\Omega }_{Ge}}{{\Omega }_{Si}})]-x\frac{{\Omega }_{Ge}/{\Omega }_{Si}}{{B}_{Ge}/{B}_{Si}}(1-x+x\frac{{\Omega }_{Ge}}{{\Omega }_{Si}})(\frac{1}{{B}_{Si}}{\frac{\partial {B}_{Si}}{\partial T}|}_{P}-\frac{1}{{B}_{Ge}}{\frac{\partial {B}_{Ge}}{\partial T}|}_{P})}{{(1-x+x\frac{{\Omega }_{Ge}/{\Omega }_{Si}}{{B}_{Ge}/{B}_{Si}})}^{2}}\end{array}$$

Similarly, the isothermal pressure derivative of the bulk modulus of the alloy, ${\partial {B}_{S{i}_{1-x}G{e}_{x}}/\partial P|}_{T}$ is expressed as

4 $$\begin{array}{l}{\frac{\partial {B}_{S{i}_{1-x}G{e}_{x}}(x)}{\partial P}|}_{T}=\frac{1+x({\Omega }_{Ge}/{\Omega }_{Si}-1)}{1+x(\frac{{\Omega }_{Ge}/{\Omega }_{Si}}{{B}_{Ge}/{B}_{Si}}-1)}{\frac{\partial {B}_{Si}}{\partial P}|}_{T}\\ -x\frac{{\Omega }_{Ge}/{\Omega }_{Si}}{{B}_{Ge}/{B}_{Si}}\frac{(1-x)(1-\frac{{B}_{Ge}}{{B}_{Si}})(1-\frac{{B}_{Si}}{{B}_{Ge}})+(1-x+x\frac{{\Omega }_{Ge}}{{\Omega }_{Si}})({\frac{\partial {B}_{Si}}{\partial P}|}_{T}-\frac{{B}_{Si}}{{B}_{Ge}}{\frac{\partial {B}_{Ge}}{\partial P}|}_{T})}{{(1-x+x\frac{{\Omega }_{Ge}/{\Omega }_{Si}}{{B}_{Ge}/{B}_{Si}})}^{2}}\end{array}$$

The coefficient of the volume thermal expansion of the alloy, ${\beta }_{S{i}_{1-x}G{e}_{x}}$ may be estimated from the following expression^30^:

5 $${\beta }_{S{i}_{1-x}G{e}_{x}}(x,T)={\beta }_{Si}\frac{1+x(\frac{{\Omega }_{Ge}}{{\Omega }_{Si}}-1)(1+\frac{\frac{\partial }{\partial T}({\frac{\partial {B}_{Si}}{\partial P}|}_{T})}{{\beta }_{Si}({\partial {B}_{Si}/\partial P|}_{T}-1)})}{1+x(\frac{{\Omega }_{Ge}}{{\Omega }_{Si}}-1)}$$

and depends only to the thermo-elastic properties of Si and the ratio of the mean atomic volumes of the two end-members. Based on Eqs 1–5, the various point defect thermodynamic parameters (*g* ^*act*^, *s* ^*act*^, *h* ^*act*^ and *υ* ^*act*^) can be calculated from Eqs 7, 9–11, if the thermo-elastic properties of the two end members are explicitly known. These relations are transferable to any binary alloy of the type A_1−*x*_B_*x*_.

### Calculation of the point defect thermodynamic parameters

The self-diffusion of Si and Ge in Si_1−*x*_Ge_*x*_ alloys has been recently studied by Kube *et al*.^2, 9^, covering almost the entire range of Ge concentration (*x* = 0.0, 0.05, 0.25, 0.45 and 0.70) and a wide temperature range (963 K–1543 K). Notably, Kube *et al*.^2, 9^ reported a non-linear behavior of activation enthalpy with Ge concentration, i.e., an upward bowing for both, Si and Ge diffusion in the alloy, with increasing *x*. In the following, we will show that these experimental findings are interpreted within the framework of the *cBΩ* thermodynamic model.

**Figure 2:**
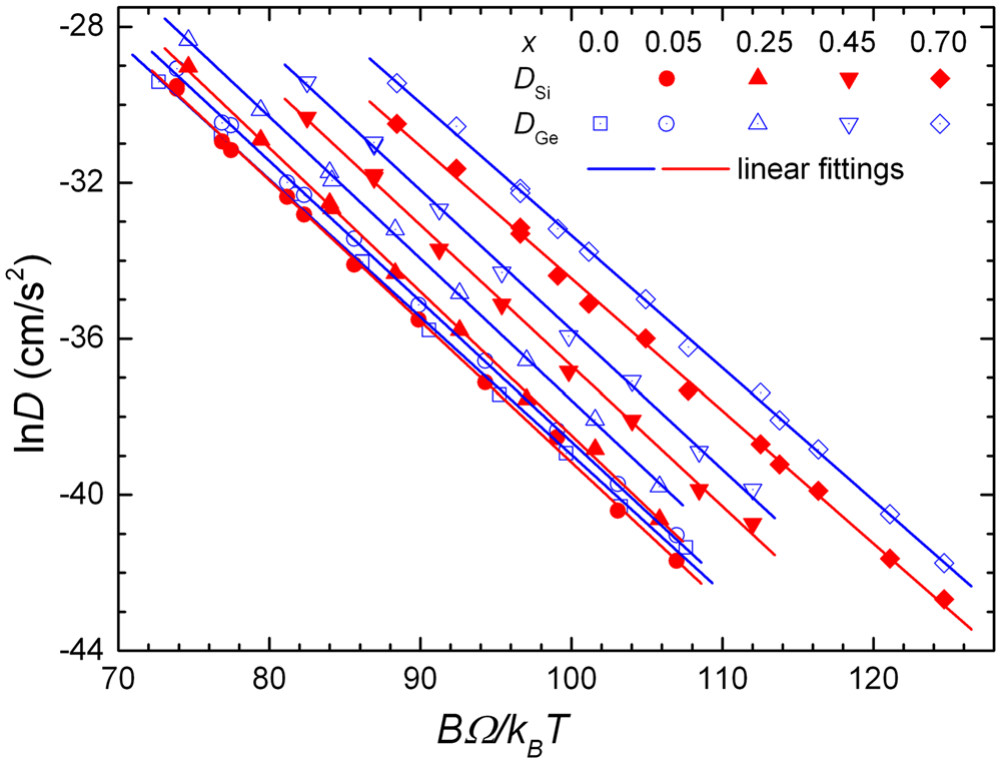
Experimental Si and Ge self-diffusion coefficients in Si_1−*x*_Ge_*x*_ alloys at different concentrations *x*, as a function of the quantity *BΩ*/*k*_*B*_ *T*. The linear behavior of the fittings (*R* ^2^ ≥ 0.997) implies the validity of the *cBΩ* model, according to Eq. 12. The experimental data were taken from refs 2 and 9.

**Figure 3:**
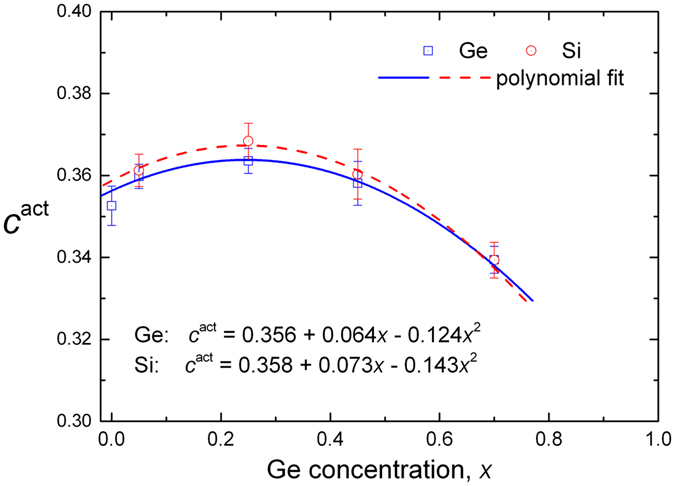
Variation of the *c* ^act^ parameter as a function of Ge concentration, *x*, for Si and Ge diffusion in Si_1−*x*_Ge_*x*_ alloys. In both cases, the data were fitted sufficiently with a 2^nd^ order polynomial (*R* ^2^ ≈ 0.96).

To proceed with the implementation of the *cBΩ* model to the Si_1−*x*_Ge_*x*_ alloy, the reported experimental values of Si and Ge self-diffusion coefficients by Kube *et al*.^2, 9^ have been plotted as a function of the quantity, *BΩ*/*k*_*B*_ *T* (see Fig. 2) as proposed in ref. 50. We recall that the bulk modulus *B* and the mean atomic volume *Ω* of the alloy have been estimated as a function of temperature and Ge concentration, according to Eqs 1 and 2. We observe that linear relations hold for both, Si and Ge diffusivities, at any concentration *x*, implying the validity of the *cBΩ* model (in accordance with Eq. 12 of the Method). The parameters *c* ^*act*^ have been estimated at each concentration from the slopes of the linear fittings and their values are shown in Fig. 3. A second order polynomial fitting has been applied to the derived values, in order to extrapolate the values of *c* ^*act*^ to the entire range of Ge concentration (0 ≤ *x* ≤ 1). Subsequently, these values are necessary to calculate the point defect thermodynamic parameters (according to Eqs 7 and 9–11 of the Method).

**Figure 4:**
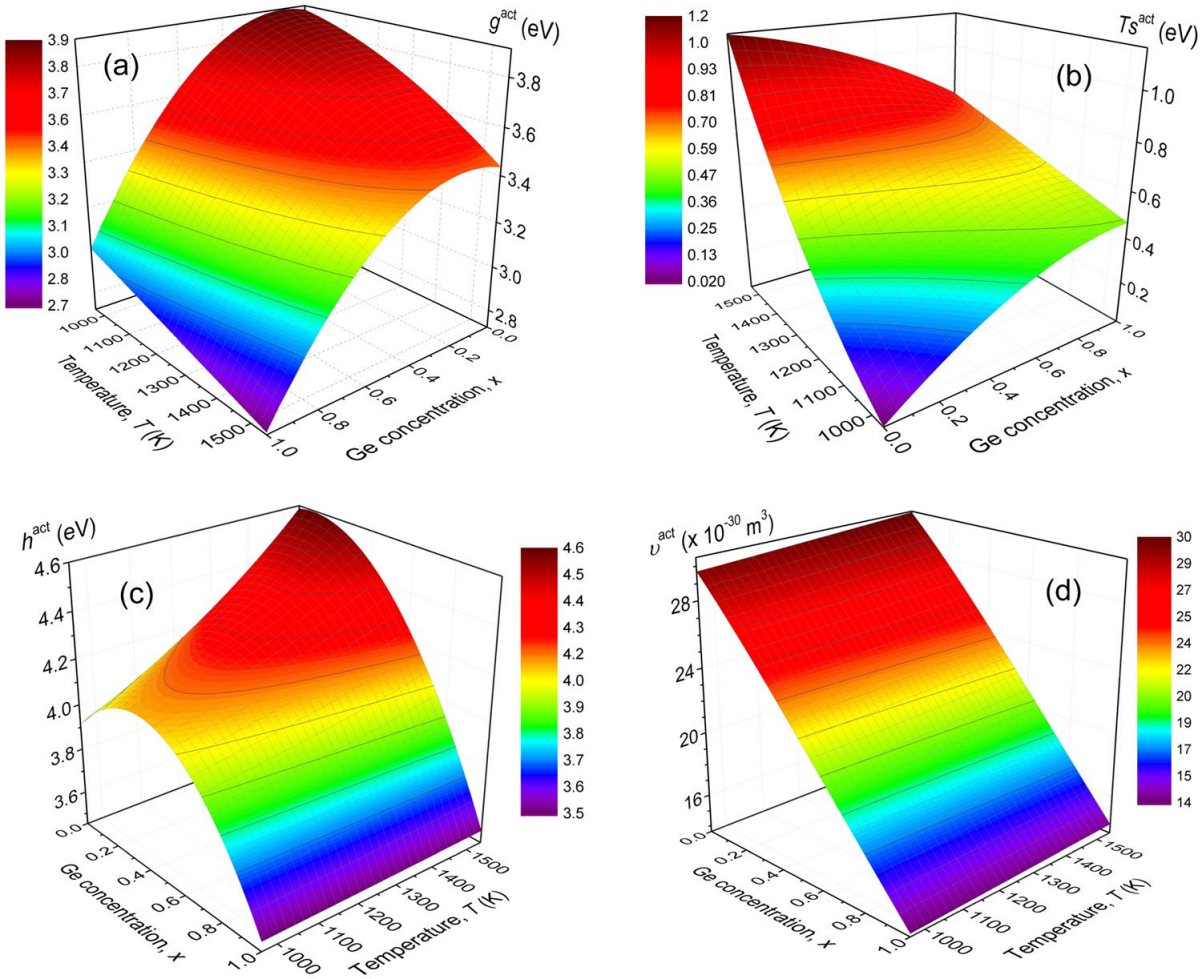
3D surface plots of the point defects thermodynamic parameters for Ge self-diffusion in Si_1−*x*_Ge_*x*_ alloys, as a function of temperature (963–1543 K) and Ge concentration, *x*, according to the *cBΩ* model. (**a**) Activation Gibbs free energy, ${g}_{Ge}^{act}$, (**b**) the energy term $T{s}_{Ge}^{act}$, (**c**) activation enthalpy, ${h}_{Ge}^{act}$ and (**d**) activation volume, ${\upsilon }_{Ge}^{act}$.

The activation Gibbs free energy, ${g}_{Ge}^{act}$ of Ge self-diffusion in Si_1−*x*_Ge_*x*_ alloys, as a function of temperature and Ge concentration, is illustrated in Fig. 4a. It is observed that, ${g}_{Ge}^{act}$ exhibits an upward bowing and a maximum value at *x* = 0.10–0.15, depending on the temperature. The effect of temperature to ${g}_{Ge}^{act}$ is also significant at the entire range of Ge concentration, resulting in a variation from 2.74 to 3.03 eV for self-diffusion in Ge (*x* = 1.0), and from 3.44 to 3.89 eV, in the case of Ge diffusion in Si (*x* = 0). Obviously, the observed variation of ${g}_{Ge}^{act}$ should have a considerable contribution to the calculated activation enthalpy.

To estimate the activation entropy, ${s}_{Ge}^{act}$ and the activation enthalpy, ${h}_{Ge}^{act}$ (through Eqs 9 and 10 of the Method), the isothermal pressure derivative of the bulk modulus of Si, ∂*B*_*Si*_/∂*P*|_*T*_ should be determined, (refer to Eq. 5) which in turn defines the thermal expansion coefficient, *β* of the alloy. To the best of our knowledge, the temperature dependence of the pressure derivative of Si bulk modulus, *B*_*Si*_ has not been determined experimentally, but it can be roughly estimated by using the Rose-Vinet universal equation of state (EoS) which states that^51, 52^

6 $$[\frac{\partial {\rm B}}{\partial P}](T,X)=\frac{4+[3{\eta }_{0}({{{\rm T}}}_{r})-1]X+{\eta }_{0}({{{\rm T}}}_{r})[{\eta }_{0}({{{\rm T}}}_{r})-1]{X}^{2}-{\eta }_{0}^{2}({{{\rm T}}}_{r}){X}^{3}}{3\{2+[{\eta }_{0}({{{\rm T}}}_{r}-1)X-{\eta }_{0}({{{\rm T}}}_{r}){X}^{2}\}}$$

where the zero subscript refers to zero (ambient) pressure, *T*_*r*_ is a reference temperature, *X* = [*Ω*_0_(*T*)/*Ω*_0_(*T*_*r*_)]^1/3^ and *η*_0_(*T*) = (3/2)(∂*B*/∂*P*|_p=0_ −1). In this way, the calculation of ${s}_{Ge}^{act}$ and ${h}_{Ge}^{act}$ becomes feasible over the entire temperature range and at any Ge concentration. Thus, the energy term, $T{s}_{Ge}^{act}$, as well as the activation enthalpy ${h}_{Ge}^{act}$ of Ge diffusion have been plotted with respect to *T* and *x* in Fig. 4b,c, respectively. A considerable change of the term $T{s}_{Ge}^{act}$ is observed for Ge diffusion in Si (*x* = 0), i.e., from 0.02 to 1.15 eV, while this change is less pronounced (0.46–0.75 eV) for Ge self-diffusion (*x* = 1). This in turn, causes a monotonic increase of $T{s}_{Ge}^{act}$ with increasing *x* at low temperatures which, however, is reversed at higher temperatures. The activation enthalpy, ${h}_{Ge}^{act}$, with respect to *x*, exhibits a maximum value (4.10 eV *at x* = 0.35) at low temperature (953 K) that is, however, shifted at lower concentrations, with increasing temperature. Finally, this maximum disappears as we approach high temperatures. At 1543 K, ${h}_{Ge}^{act}$ decreases from 4.59 eV to 3.50 eV, with increasing Ge concentration, exhibiting an upward bowing. This non-linear behavior is in good agreement with the experimental values of activation enthalpy of diffusion reported by Kube *et al.* ^2, 9^. Specifically, they reported a variation from 4.83 to 3.13 eV for Ge diffusion, which has been described by a quadratic correction term in Vegard’s law i.e., *Q*(*x*) = (1 − *x*)*Q*(0) + *xQ*(1) + *x*(1 − *x*)*Θ*, with *Θ* denoting the bowing parameter. This empirical description arises effortlessly within the framework of the *cBΩ* model and may be attributed to the diversification of the bulk properties of Si and Ge. Specifically, it has been recently reported^33^ that the non-linear temperature dependence of activation enthalpy and activation entropy of self-diffusion in Si, which has been experimentally measured by Kube *et al*.^53^, can be explained in terms of the *cBΩ* model, by considering the non-linear anharmonic behavior of the isothermal bulk modulus of Si^48^. This peculiar behavior of diffusion in Si is clearly observed in Fig. 4c where ${h}_{Ge}^{act}$ in Si (*x* = 0) varies non-linearly with temperature from 3.91 to 4.59 eV. In contrast, at high Ge content (*x* > 0.6), no temperature dependence of ${h}_{Ge}^{act}$ is observed, due to the linear variation of the bulk modulus of Ge at this temperature range, in agreement with a previous study of self-diffusion in Ge^32^.

Finally, the temperature and concentration dependence of the activation volume, ${\upsilon }_{Ge}^{act}$ is illustrated in Fig. 4d. We observe that the temperature dependence of ${\upsilon }_{Ge}^{act}$ is negligible all over the concentration range of Ge, while a considerable variation of ${\upsilon }_{Ge}^{act}$ occurs with increasing concentration. Specifically, at 963 K, ${\upsilon }_{Ge}^{act}$ decreases from 29.7 Å^3^ (at *x* = 0) to 14.2 Å^3^ for Ge self-diffusion (*x* = 1). These values correspond to (1.48 ± 0.07)*Ω*_*o*,*Si*_ and (0.62 ± 0.06)*Ω*_*o*,*Ge*_, respectively, in agreement with previous calculations of self-diffusion in Si and Ge^32, 33, 54^. The sign and magnitude of the activation volume provides evidence on the diffusion mechanism, i.e., vacancy (*V*) or self-interstitial (*I*), through the relation ${\upsilon }_{V,I}^{act}=\pm {{\Omega }}_{o}+{\upsilon }_{V,I}^{r}+{\upsilon }_{V,I}^{m}$, where the positive sign of *Ω*_*o*_ refers to *V* and the negative to *I* formation, ${\upsilon }_{V,I}^{r}$ is the relaxation volume around the point defect (*V* or *I*) and ${\upsilon }_{V,I}^{m}$ is the corresponding migration volume^33, 55^. In the present study, the positive sign of ${\upsilon }_{Ge}^{act}$ and the range of the calculated values indicate that the vacancy mechanism is prevalent throughout the temperature range considered. Ignoring the negligible effect of temperature to ${\upsilon }_{Ge}^{act}$, we observe that Vegard’s law describes in a good approximation the activation volumes of Si_1−*x*_Ge_*x*_ alloys with Ge concentration, *x*, i.e., ${\upsilon }_{Ge}^{act}(x)=(1-x){\upsilon }_{o,Si}^{act}+x{\upsilon }_{o,Ge}^{act}$, where ${\upsilon }_{o,Si}^{act}$ and ${\upsilon }_{o,Ge}^{act}$ refer to the activation volumes of the end members.

According to the *cBΩ* model, the point defect thermodynamic parameters (see Eqs 7 and 9–11) are functions of the bulk properties of the alloy and thus, for diffusion of Ge or Si in Si_1−*x*_Ge_*x*_ alloys, the only factor that affects further the results for different diffusants is the parameter *c* ^*act*^. Since the values of *c* ^*act*^ are quite similar for both, Ge and Si diffusion, we do not expect substantial variations of *g* ^*act*^, *h* ^*act*^, *s* ^*act*^ and *υ* ^*act*^ for the case of Si diffusion, as compared to Ge diffusion in the Si_1−*x*_Ge_*x*_ alloys (see Supplementary Information). The latter is in agreement with the similar experimental values of activation enthalpy, reported by Kube *et al*. for Ge and Si diffusion in Si_1−*x*_Ge_*x*_ alloys^2, 9^.

### Overcoming limitations and prospects of the *cBΩ* model

In order to apply the *cBΩ* thermodynamic model to estimate various point defect parameters, the thermo-elastic properties of the two end members of the binary alloy should be known as a function of temperature and/or pressure. However, these bulk properties are not always known from experimental or theoretical studies. Even for “simple” well studied binary systems such the Si_1−*x*_Ge_*x*_ alloy of the present study, the derivative of the bulk modulus has not been defined experimentally and it has been approximated by using the Rose-Vinet universal EoS^51, 52^. Furthermore, the temperature derivative of ∂*B*/∂*P*|_*T*_ (see Eq. 5) may be difficult to find in the literature, however, it can be estimated via the approximation, ∂*B*/∂*P*|_*T*_ ≈ ∂*B* ^*S*^/∂*P*|_*T*_ + 2*T* *βγ*, where *γ* is the Grüneisen constant and *B* ^*S*^ is the adiabatic bulk modulus^30^. By disregarding small temperature dependencies of ∂*B* ^*S*^/∂*P* and *γ*, the aforementioned unknown quantity is finally approximated with the expression 2*γ*(*β* + T(∂*β*/∂*T*|_*P*_) which contains easily accessible terms^30^.

The application of the *cBΩ* model in binary alloys will depend upon the availability of parameters that can be calculated either by experiment and/or DFT calculations. As it is discussed above, approximations can also be used to overcome the lack of certain parameters thus the calculation of the point defect parameters becomes feasible.

### Summary

In the present study, the self-diffusion of Si and Ge in Si_1−*x*_Ge_*x*_ alloys has been investigated in the framework of the *cBΩ* thermodynamic model, which allows the calculation of point defect thermodynamic parameters from the bulk properties of the alloy. The consideration of a wide temperature range and the whole composition range of Si_1−*x*_Ge_*x*_ in conjunction with the excellent agreement of the calculated values as compared to the available experimental data demonstrates the efficacy of the approach.

Here we demonstrate how the *cBΩ* model can become applicable and provide valuable information for the self- or hetero-diffusion and point defect thermodynamic parameters in binary alloys. This method in conjunction with experiment and/or advanced modeling techniques can be employed in numerous systems including for example solid solution MAX phases, nuclear materials, ternary semiconductors and disordered ionic conductors^56–58^.

## Methods

In the context of the *cBΩ* model^28–30^, the activation Gibbs free energy *g* ^*act*^ due to the formation and migration of a point defect in a solid is related to its elastic and expansion properties via:

7 $${g}^{act}={c}^{act}B{\rm{\Omega }}$$

In equation (7), *B* is the isothermal bulk modulus, Ω stands for the mean atomic volume, while *c* ^*act*^ is a dimensionless constant which is independent of temperature and pressure but depends on the diffusion mechanism (i.e., vacancy or interstitial) and the host material. The diffusion coefficients *D* of a single diffusion mechanism exhibiting an Arrhenius behavior are:

8 $$D(T,P)=fg{{\rm{a}}}_{o}^{2}\nu {e}^{-{c}^{act}B\Omega /{k}_{B}T}$$

where *f* is the diffusion correlation factor which depends on the diffusion mechanism and the crystal structure, *g* is a geometrical factor, a_*o*_ is the lattice parameter, *ν* is the attempt frequency and *k*_*B*_ is Boltzmann’s constant.

The connection of point defect thermodynamic parameters, such as the activation entropy *s* ^*act*^ and the activation enthalpy *h* ^*act*^, to the elastic and expansion properties of the bulk material, which is in essence the *cBΩ* model, is expressed through^28, 29^:

9 $${s}^{act}=-{\frac{\partial {g}^{act}}{\partial T}|}_{P}=-{c}^{act}\Omega \{{\frac{\partial B}{\partial {\rm T}}|}_{P}+\beta B\}$$

and

10 $${h}^{act}={g}^{act}+T{s}^{act}={c}^{act}\Omega \{B-T\beta B-T{\frac{\partial B}{\partial {\rm T}}|}_{P}\}$$

where *β* is the volume thermal expansion coefficient which depends on temperature and pressure. The above important thermodynamic parameters (i.e., *s* ^*act*^ and *h* ^*act*^) are determined experimentally and thus equations (9) and (10) can be used to validate the *cBΩ* model and calculate the activation enthalpy and activation entropy when there is insufficient experimental diffusion data.

The activation volume, *υ* ^*act*^ in terms of the *cBΩ* model^30^, is expressed as

11 $${\upsilon }^{act}={\frac{\partial {g}^{act}}{\partial P}|}_{T}={c}^{act}\Omega \{{\frac{\partial B}{\partial P}|}_{T}-1\}$$

For the proper implementation of the *cBΩ* model according to equations (7–11), the estimation of the constant *c* ^*act*^ is necessary. At zero temperature, *g* ^*act*^ equals to ${h}_{o}^{act}$ and thus, in principle, the constant *c* ^*act*^ has the value ${h}_{o}^{act}/{B}_{o}{{\rm{\Omega }}}_{o}$, where the subscripts refer to *T* = 0 K^27^. The most reliable method of the calculation of *c* ^*act*^ is the mean value method which is applicable when experimental diffusion data are available over a broad temperature or pressure range^22, 23, 30, 32^. By taking the natural logarithm of both sides in equation (8), we obtain:

12 $$lnD=\,\mathrm{ln}(fg{{\rm{a}}}_{0}^{2}\nu )-{c}^{act}\frac{B\Omega }{{k}_{B}T}$$

According to equation (12), a linear dependence of *lnD* versus the quantity *BΩ*/*k*_*B*_ *T* indicates the validity of the *cBΩ* model as concern a single diffusion mechanism, and the constant *c* ^*act*^ arises directly from the slope of the linear fitting.

## Electronic supplementary material

Supplementary Information
