# Supplementary material for: Composition and temperature dependence of self-diffusion in Si1−xGex alloys
Source: Sci Rep. 2017 May 2;7:1374. doi: 10.1038/s41598-017-01301-6 (PMC5430970; doi:10.1038/s41598-017-01301-6)
Supplement: Supplementary file 1 — Supplementary Information [file 41598_2017_1301_MOESM1_ESM.pdf]

Composition and temperature dependence of self-diffusion in $Si_{1-x}Ge_x$ alloys

V. Saltas,[a,1] A. Chroneos,[b,c,1], F. Vallianatos[a]

[a]*School of Applied Sciences, Technological Educational Institute of Crete, Greece.*
[b]*Department of Materials, Imperial College London, London SW7 2AZ, United Kingdom.*
[c]*Faculty of Engineering, Environment and Computing, Coventry University, Priory Street, Coventry CV1 5FB, United Kingdom.*

SUPPLEMENTARY INFORMATION

Deviation from Vegard's law in $Si_{1-x}Ge_x$ alloy

In the case of the $Si_{1-x}Ge_x$ alloy, a deviation from Vegard's law has been proposed in order to describe the lattice constant of the alloy at 300 K with respect to Ge concentration, which is expressed through the following parabolic equation[41,42]

$$a(x) = 5.6575 - 0.2530x + 0.0266x^2 \quad \text{(S1)}$$

Based on the above equation, the mean atomic volume has been calculated and is depicted in Fig. S1. The maximum deviation of the calculated values from the linear aproximation that is used in the present study (see Eq. 1) is about 4%.

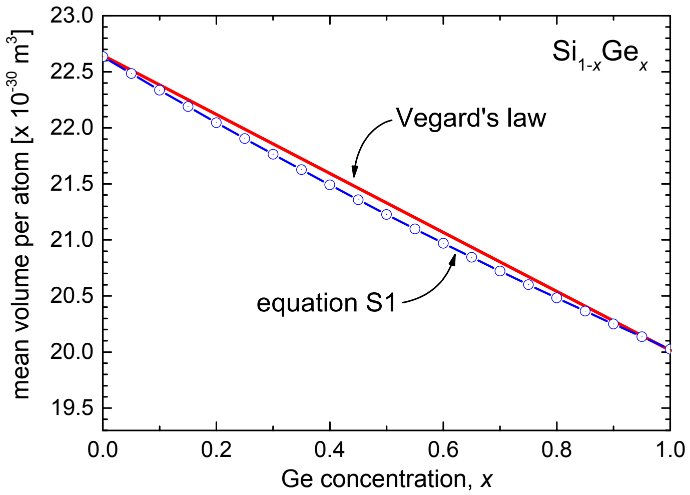

Fig. S1 Mean volume per atom of $Si_{1-x}Ge_x$ alloy, as a function of Ge concentration, *x*. The red line represents the linear variation between the two end-members, according to Eq. 1.

[1] Corresponding authors, e-mail: a) vsaltas@chania.teicrete.gr; b) alexander.chroneos@imperial.ac.uk

Point defect thermodynamic parameters of Si self-diffusion in $Si_{1-x}Ge_x$ alloys

In the case of Si self-diffusion in $Si_{1-x}Ge_x$ alloys, the activation Gibbs free energy g_{Si}^{act}, the activation entropy s_{Si}^{act}, the activation enthalpy h_{Si}^{act}, and the activation volume υ_{Si}^{act} have been calculated according to the *cBΩ* model and the results are shown in Figure S2. The trends of these point defect parameters, as well as their absolute values are quite similar with the corresponding values in the case of Ge diffusion (see Figs. 4-7). Slightly higher values for the activation enthalpy of Si compared to Ge are observed, in agreement with the reported results [2,9].

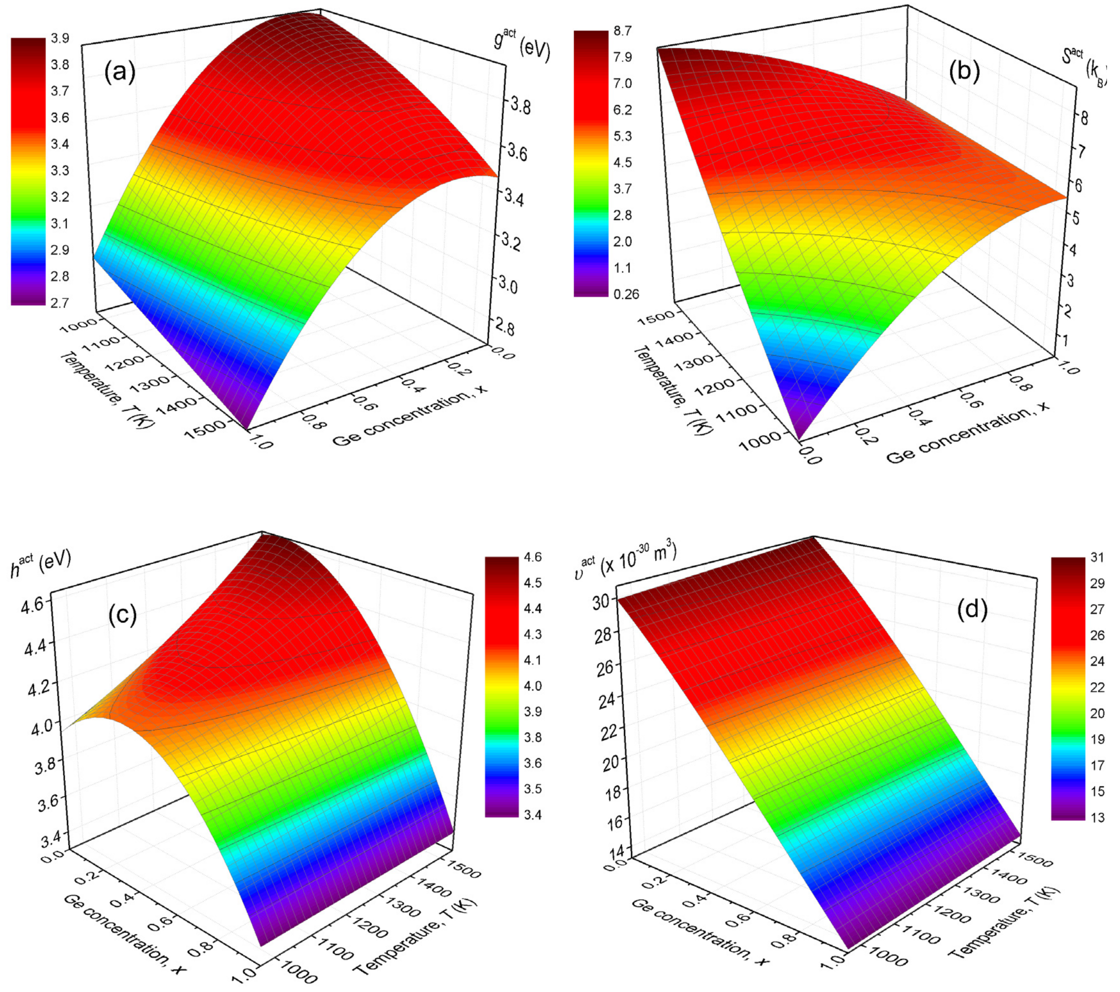

Fig. S2 Point defect thermodynamic parameters of Si self-diffusion in $Si_{1-x}Ge_x$ alloys, as a function of temperature (963 K-1543 K) and Ge concentration ($0 \leq x \leq 1$). (a) Activation Gibbs free energy, (b) activation entropy, (c) activation enthalpy and (d) activation volume.
